# Preparation and Characterization of a Novel Soy Protein Isolate-Sugar Beet Pectin Emulsion Gel and Its Application as a Multi-Phased Nutrient Carrier

**DOI:** 10.3390/foods11030469

**Published:** 2022-02-05

**Authors:** Minghao Zhang, Lijun Yin, Wenjia Yan, Chong Gao, Xin Jia

**Affiliations:** Beijing Key Laboratory of Functional Food from Plant Resources, College of Food Science and Nutritional Engineering, China Agricultural University, Beijing 100083, China; zmhao@cau.edu.cn (M.Z.); ljyin@cau.edu.cn (L.Y.); sevenyan@cau.edu.cn (W.Y.); eve_bigg@sina.com (C.G.)

**Keywords:** SPI-SBP emulsion gel, duo-inducing, co-loading

## Abstract

Emulsion gel, a novel oral delivery carrier, provides the possibility to co-load hydrophilic and lipophilic nutrients simultaneously. In this study, duo-induction methods of laccase and glucono-δ-lactone (L&GDL) or laccase and transglutaminase (L&MTG) were used to prepare the soy protein isolate-sugar beet pectin (SPI-SBP) emulsion gel. The textural data of the emulsion gel was normalized to analyze the effect of different induction methods on the gel property of the SPI-SBP emulsion gels. The characterization studies showed the structure of L&MTG emulsion gel was denser with a lower swelling ratio and reduced degree of digestion, compared with L&GDL emulsion gel. Moreover, the release profiles of both β-carotene and riboflavin co-loaded in the SPI-SBP emulsion gels were correlated to the digestion patterns of the gel matrix; the controlled-release of encapsulated functional factors was regulated by a gel network induced by different induction methods, mainly due to the resulting porosity of the structure and swelling ratio during digestion. In conclusion, SPI-SBP emulsion gels have the capability of encapsulating multiple functional factors with different physicochemical properties.

## 1. Introduction

Designing a bio-degradable and safe delivery system for nutrient delivery has attracted much attention in recent years [1]. Traditional hydrogels have limited applications due to their inability to encapsulate lipophilic nutrients. Therefore, emulsion gels have been developed to increase the delivery capacity of lipophilic biologically active nutrients and to further expand the application of hydrogels. Emulsion gels have a three-dimensional network structure consisting of oil droplets within a hydrogel matrix [2]. The presence of the oil droplets does not only improve the textural properties of the hydrogel matrix, but also allows for the delivery of lipophilic biomolecules [3]. Moreover, due to the coexistence of water and oil phases, the emulsion gels can also act as an ideal multi-phased nutrient carrier for functional biomolecules with varied solubility and physicochemical properties [4]. Functional factors with different solubility can be loaded within the same carrier simultaneously, which provides the possibility for functional factors to play the synergistic action at the target.

Food-grade emulsion gels are usually constructed by biomacromolecules such as proteins and polysaccharides. Proteins are amphiphilic and are therefore often used as an emulsifier in an oil-water system. Although research on emulsion gels fabricated by whey protein isolate (WPI) [5], soy protein isolate (SPI) [6] and casein [7] have been reported, there have been many reported cases associated with protein flocculation and poor stability during food processing and storage. Polysaccharides have also been used as the matrix of food emulsion gel systems and usually exhibit enhanced rigidity, such as gellan gum [8], sodium alginate [9], and pectin [10]. Compared with the single protein or polysaccharide-matrix emulsion gel, the network structure of emulsion gels formatted by proteins and polysaccharides can be regulated by the ratio between different biomacromolecules, and the ability of the gels to control-release functional factors in the gastrointestinal tract is also improved [11].

Protein-polysaccharide emulsion gels can be fabricated through methods such as physical alterations, ionic strength, pH, and applications of enzymes [12]. For low-temperature gels, pH and enzyme alterations are the two main induction methods. Adjusting pH is one of the common methods used in the food industry due to convenience in the application. It is achieved by reducing pH until the system reaches the isoelectric point (pI) of the protein, to allow for protein aggregation and precipitation. For example, in the addition of glucono-δ-lactone (GDL), SPI neutralized by acidification reached the pI, which led to the formation of a protein gel network [13]. Additionally, the enzymatic induction method also has been widely used in the preparation of food-grade emulsion gels due to high substrate specificity, efficiency, and stability. For example, transglutaminase (MTG) catalyzes acyl transfer reactions and is mostly used to catalyze protein cross-linking gels [14]. Another enzyme commonly used for emulsion-gel induction is laccase. Laccase induces gel formation mainly through catalyzing the cross-linking reaction between tyrosine residue in proteins or ferulic acid from polysaccharides [15]. Laccase has been widely applied in various food processes such as bioremediation, beverage processing, sugar beet pectin gelation and baking, to improve food sensory parameters [16]. Moreover, laccase can be used in combination with other methods (such as heating, pH, and other enzymes) to improve the textural properties of food through catalyzed gel formation. In a previous study, laccase catalysis and thermal co-treatment were used to form SPI-SBP gel [17], but most of nutrients in food would lose their functional activity due to thermal treatment. To the best of our knowledge, SPI-SBP gels induced by laccase in the absence of high heat has yet to be reported.

The current study aims to prepare a novel SPI-SBP emulsion gel as a multi-phased carrier to co-encapsulate both hydrophilic and lipophilic nutrients. The effect of different duo-induction methods using laccase in combination with GDL or MTG on the structural properties of SPI-SBP emulsion gel was evaluated. The digestive properties of the selected SPI-SBP emulsion gels for the subsequent release rate of bioactive substances were also measured. The establishment of a relationship among the gelation mechanisms, texture, microstructure and digestive profiles was attempted. The current study broadens the application of SPI-SBP emulsion gels as a multi-phased nutrient carrier to encapsulate thermal-sensitive substances.

## 2. Materials and Methods

### 2.1. Materials

Soy protein isolate (SPI) was supplied by Shandong Yuwang Ecological Food Industry Co., Ltd. (Shandong, China). Sugar beet pectin (SBP) was purchased from Herbstreith & Fox KG (Elmsford, Germany). Medium-chain triglycerides (MCT) was obtained from Guangzhou Yeshang trade Co., Ltd. (Guangdong, China). Laccase (≥0.5 U/mg) was provided by Sigma-Aldrich Co. Ltd. (St. Louis, MI, USA). Glucono-δ-lactone (GDL) was purchased from Beijing Solarbio Science & Technology Co., Ltd. (Beijing, China). Transglutaminase (MTG) (≥1.5 U/mg) was supplied by Kuer Chemical Technology Co., Ltd. (Beijing, China). All other chemicals were of analytical grade.

### 2.2. Preparation of SPI-SBP Emulation Gel

SBP (2.0%, *w*/*w*) was dissolved in an SPI solution (6.0%, *w*/*w*) and the mixture was stirred for 4 h at room temperature. After being stored at 4 °C overnight, the SPI-SBP solution was heated at 85 °C for 15 min before being cooled down to room temperature immediately. Then, 20% MCT (*w*/*w*) was added to the SPI-SBP solution, and the mixture was mixed with a high-speed shear machine (T25 basic, IKA, Staufen, Germany) at 10,000 r/min for 3 min, followed by homogenization five times at 30 MPa with a homogenizer (EmusiFlex-C5, Avestin, Ottawa, ON, Canada) to obtain the SPI-SBP emulsion. The emulsion was mixed with GDL (0.3–1.8%, *w*/*w*) and stirred for 1 min to yield the GDL SPI-SBP emulsion, or mixed with MTG (5–30 U/g protein) to obtain the MTG SPI-SBP emulsion. Next, a 20 U/g substrate of laccase was added into GDL or MTG SPI-SBP emulsion and stirred for 1 min to obtain L&GDL or L&MTG SPI-SBP emulsion. A control emulsion was prepared with 20 U/g substrate of laccase with SPI-SBP emulsion to illustrate the advantages of duo-induced emulsion gels in gel properties and functional factor delivery. Finally, the laccase, L&GDL, or L&MTG emulsion gels were prepared after heating the L&GDL or L&MTG emulsion at 40 °C for 4 h. All prepared gels were settled at 4 °C overnight for the subsequent experiments.

### 2.3. Texture Profile Analysis (TPA) of SPI-SBP Emulsion Gel

For texture analysis, two down-pressure tests using a TMS-Pro Texture Analyzer (Food Technology Corp., Sterling, VA, USA) and the 20 mm diameter P/20a cylindrical probe were performed to measure the textural properties of the SPI-SBP emulsion gel. The TPA parameters were as follows: the pressing height was 25% of the sample height, and the test speed was 60 mm/min. The TPA curves were recorded and the parameters (hardness, springiness, and cohesiveness) were calculated using the software provided by the Texture Analyzer [17].

### 2.4. Minkowski Distance Normalization

The original texture parameters (hardness, springiness, and cohesiveness) were normalized based on the Minkowski distance to obtain the comprehensive property index (CPI) of all samples [18]. The correlation between three texture parameters (hardness, springiness, and cohesiveness measured as described in Section 2.3) and the CPI of each sample was analyzed using a Pearson correlation analysis. The accuracy and reliability of the model were evaluated by the obtained correlation coefficient. A regression analysis was performed to analyze the relationship between the original texture data and CPI.

### 2.5. Microstructure of SPI-SBP Emulsion Gel

The previously obtained SPI-SBP emulsion gel was freeze-dried before being observed on a scanning electron microscope (SEM, Hitachi S-480, Tokyo, Japan) at a voltage of 15 kV.

Based on a previously published method [19], SBP was labeled with fluoresceine isothiocyanate (FITC), and the other preparation processes of SPI-SBP emulsion were the same as described in Section 2.2. Then, 20 μL of 0.2% (*w*/*v*) rhodamine B and 0.1% (*w*/*v*) Nile Red mixed staining solution was added into 2 g of the prepared SPI-SBP emulsion, after which the gelling agents were added according to the method described in Section 2.2 to obtain L&GDL or L&MTG SPI-SBP emulsion and the gelation process was finished on a glass slide. The confocal laser scanning microscope (CLSM) was used to observe SBP labeled with FITC at 488 nm, SPI labeled with Nile Red at 553 nm, and MCT labeled with rhodamine B at 633 nm.

### 2.6. Swelling Properties Measurement of SPI-SBP Emulsion Gel

To assess the swelling property of the prepared emulsion gel, fresh prepared SPI-SBP emulsion gel was soaked in a 0.02% sodium azide solution (to prevent microbial spoilage) for 48 h [20], and the gel swelling ratio was calculated based on the following equation

(1) $$\mathrm{Swelling}\;\mathrm{rate}\;\left(\%\right)=\frac{w_{2}-w_{1}}{w_{1}}\times 100\%$$

where w_1_ is the weight of the fresh emulsion gel and w_2_ is the weight of the emulsion gel after soaking.

### 2.7. In Vitro Simulated Digestion Test

The SPI-SBP emulsion gels were cut into 0.2 cm × 0.2 cm × 0.2 cm cubes. These cubes were firstly incubated in simulated saliva fluid (SSF, pH 7.0) at 37 °C for 5 min, and the ratio of gel cubes to SSF of 50:50 (*w*/*v*) was targeted. After that, the same volume of simulated gastric fluid (SGF, pH 2.0, 37 °C) was added to the oral mixture. Following this, 2000 U/mL of porcine pepsin (EC 3.4.23.1) was added to the mixture. The mixture was incubated at 37 °C for 120 min. After gastric digestion, the gastric chyme was mixed with the same volume of simulated intestinal fluid (SIF, pH 7.0, 37 °C). The activity of trypsin (EC 3.4.21.4) and pancreatic lipase (EC 3.1.1.3) was 100 U/mL and 2000 U/mL in the final mixture, and the final concentration of bile salts was 10 mmol/L. The final mixture was incubated at 37 °C for 120 min. All incubating processes were performed in a 37 °C water bath shaker with a speed of 120 rpm [21].

#### 2.7.1. Free Amino Acid (FAA) Determination

The determination of FAA was based on the method published by Rui [22]. After in vitro digestion, the digestive mixtures of SPI-SBP emulsion gels were centrifuged at 8000× *g* for 10 min using a bench-top centrifuge (MH2100RA, Merrick Instruments Co., Ltd., Shanghai, China). Next, 10 μL of the supernatant was added into a 96-well microtiter plate, and 100 μL of ninhydrin reagent (20 mg/mL) dissolved in acetate buffer (pH 5.5, contained 2.5 mg/mL SnCl_2_) was added into the mixture; the resulting microtiter plate was heated at 104 °C for 10 min. The absorbance was measured at 575 nm using a microplate reader (Multiskan FC, Thermo Fisher Scientific, Shanghai, China), and glycine standards were used to calculate the amount of FAA in the samples.

#### 2.7.2. Free Fatty Acid (FFA) Determination

In this study, a titration method reported by Sarkar [23] was used for the determination of FFA during digestion. After being digested in SSF and SGF, the SPI-SBP emulsion gels were transformed into SIF and a pH automatic titrator was used to titrate FFA released from the SPI-SBP emulsion gels in SIF. During titration, the pH of the system was kept at 7.0. The volume of 0.3 M NaOH used for neutralization of the system was recorded. The percentage of FFA released was calculated using the following equation

(2) $$\mathrm{FFA}\;\left(\%\right)=\frac{\left(V_{\mathrm{NaOH}}-V_{\mathrm{blank}}\right)\times C_{\mathrm{NaOH}}\times M_{\mathrm{MCT}}}{w_{\mathrm{MCT}}}\times 100\%$$

where $V_{\mathrm{NaOH}}$ (mL) was the volume of titrant used; $V_{\mathrm{blank}}$ (mL) was the volume of the MCT-free SBP-SPI emulsion gel which was used as a blank sample in the experiment; $C_{\mathrm{NaOH}}$ (mol/L) was the concentration of NaOH; $M_{\mathrm{MCT}}$ was the molecular weight of MCT; and $w_{\mathrm{MCT}}$ (g) was the weight of MCT added to the digestive fluid.

### 2.8. Characterization of SPI-SBP Emulsion Gel Co-Loading Riboflavin and Β-Carotene

#### 2.8.1. Preparation of SPI-SBP Emulsion Gel Co-Loading System

Based on Section 2.2, 0.6 mg/mL riboflavin was added to the SPI-SBP solution, and the preparation of aqueous phase of the emulsion was identical to the process described above. The β-carotene (0.2% (*w/w*)) was dissolved in MCT at 45 ℃ to prepare the emulsion oil phase. Steps to prepare SPI-SBP emulsions and emulsion gels (laccase, L&GDL, and L&MTG SPI-SBP emulsion gel) co-loading systems were carried out following the steps described in Section 2.2.

#### 2.8.2. Determination of Embedding Efficiency (EE) in SPI-SBP Gel Delivery System

Freshly prepared emulsion samples and emulsion-gel samples loaded with nutrients were freeze-dried. A total of 0.5 g of each freeze-dried sample was soaked in 10 mL DI water for 24 h to release the embedded nutrients. Following this, 10 mL of n-hexane was added to the mixture, which was centrifuged at 8500× *g* for 10 min. The n-hexane phase was collected and the absorbance was measured at 450 nm using a microplate reader (Multiskan FC, Thermo Fisher Scientific, Shanghai, China). The β-carotene standards were used to calculate the amount of β-carotene in the hexane layer [24]. The amount of riboflavin in the residual aqueous layer after centrifugation was also measured at 450 nm, for which the calculations were based on the riboflavin calibration curves [25,26]. The EE of nutrients was calculated using Equation (3)

(3) $$\mathrm{EE}\left(\%\right)=\frac{w_{1}-w_{2}}{w_{1}}\times 100\%$$

where EE (%) is the embedding efficiency of nutrients (β-carotene in n-hexane phase or riboflavin in water phase), w_1_ (mg) is the total amount of nutrients, w_2_ (mg) is the amount of nutrients in the supernatant.

#### 2.8.3. Determination of Release Rate in SPI-SBP Delivery System during Digestion In Vitro

The co-loading systems of the SPI-SBP emulsion and emulsion gels (laccase, L&GDL, and L&MTG SPI-SBP emulsion gel) were digested based on the digestive procedure described in Section 2.7. All samples in every digestive phase were adjusted to pH 7.0 before measurement. The amount of riboflavin and β-carotene was determined according to Section 2.8.2. The release rate (%) of nutrients was calculated using Equation (4)

(4) $$\;\mathrm{Release}\;\mathrm{rate}\left(\%\right)=\frac{w_{1}-w_{2}}{w_{1}}\times 100\%$$

where w_1_ (mg) is the total amount of nutrients, w_2_ (mg) is the amount of nutrients in the supernatant.

### 2.9. Statistics and Analysis

SPSS Statistics 26.0 software was used to analyze the data, and one-way analysis of variance (ANOVA) was used to determine the significant difference between each test (*p* < 0.05). Matlab 2016 software was used to analyze and normalize the textural data statistically and to perform a regression analysis as well as Pearson correlation analysis. The experimental data were processed using Origin 2019 software, and the results were expressed in means ± standard deviation. All experiments were conducted in triplicate.

## 3. Results and Discussion

### 3.1. Textural Properties of SPI-SBP Emulsion Gels

**Figure 1:**
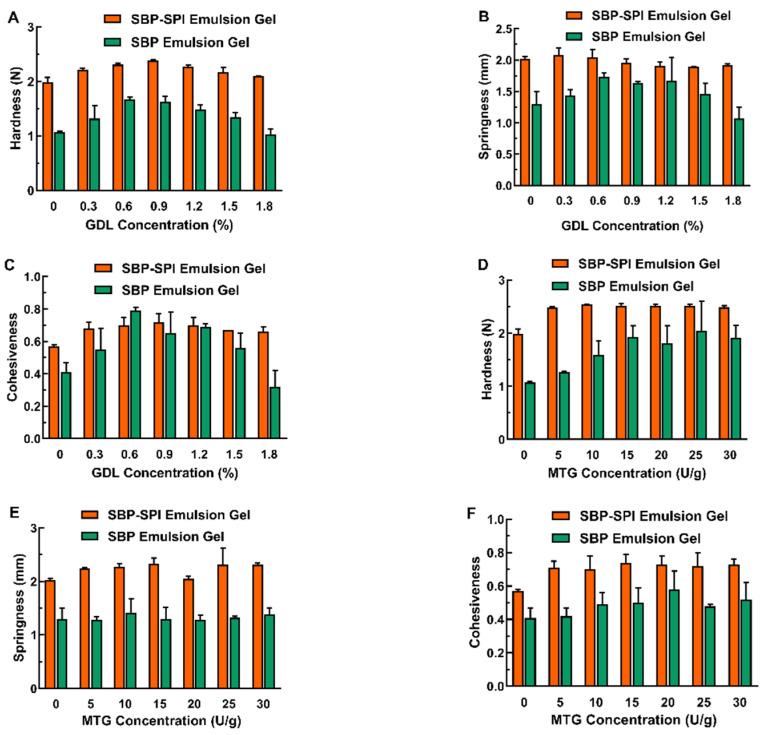
The hardness (**A**,**D**), springiness (**B**,**E**), and cohesiveness (**C**,**F**) of SPI-SBP and SBP emulsion gels induced by L&GDL (**A**–**C**) and L&MTG (**D**–**F**).

The textural properties were characterized by TPA, as shown in Figure 1. The L&GDL induction method showed improved textural properties of SPI-SBP emulsion gel than that of laccase single-induced SPI-SBP emulsion gel and L&GDL SBP emulsion gel (Figure 1A–C). As displayed in Figure 1A, as the GDL concentration increased, the hardness of SPI-SBP emulsion gel improved. In SPI-SBP emulsion, acidification caused by GDL in the system continued to approach the protein pI, which eventually caused SPI to form a gel network [4]. When the concentration of GDL continued to increase, the pH of the emulsion decreased faster, until it eventually reached “over-acidification” as the pH of the emulsion deviated from the pI (Figure S1). At this point, proteins became positively charged, and the gel strength decreased as the repulsive force between SPI molecules became more pronounced. The SBP network in both SPI-SBP and SBP emulsion gel was induced by laccase and the optimum pH for laccase activity ranged from 4.0–5.0 [15]. Thus, decreasing the pH of the emulsion also induced the laccase to form a SBP gel network at a low GDL concentration. Nevertheless, when the pH of the emulsion was far from the optimum pH for laccase activity, it resulted in a decrease in the strength of the SBP network, leading to a weakening of the SPI-SBP and SBP emulsion gel.

On the other hand, the hardness (Figure 1D), springiness (Figure 1E), and cohesiveness (Figure 1F) of the SPI-SBP emulsion gel induced by L&MTG were better than the SBP emulsion gel induced by L&MTG. In the L&MTG SPI-SBP emulsion gel, the SBP network skeleton was formed under the action of laccase. Within the same system, MTG promoted the formation of the SPI network, and the double network formed by SPI and SBP increased the mechanical strength of the SPI-SBP emulsion gel. The results also show that compared with the SBP emulsion gel induced by laccase alone, the addition of MTG enhanced the hardness and cohesiveness of the SBP emulsion gel. This is because SBP usually contains 1.5–4.5% protein, and under the action of MTG, lysine residue within proteins can cross-link to form covalent bonding between SBP intra- and inter-molecularly [27], resulting in a denser SBP network.

### 3.2. Normalized Analysis of Texture Indexes

**Figure 2:**
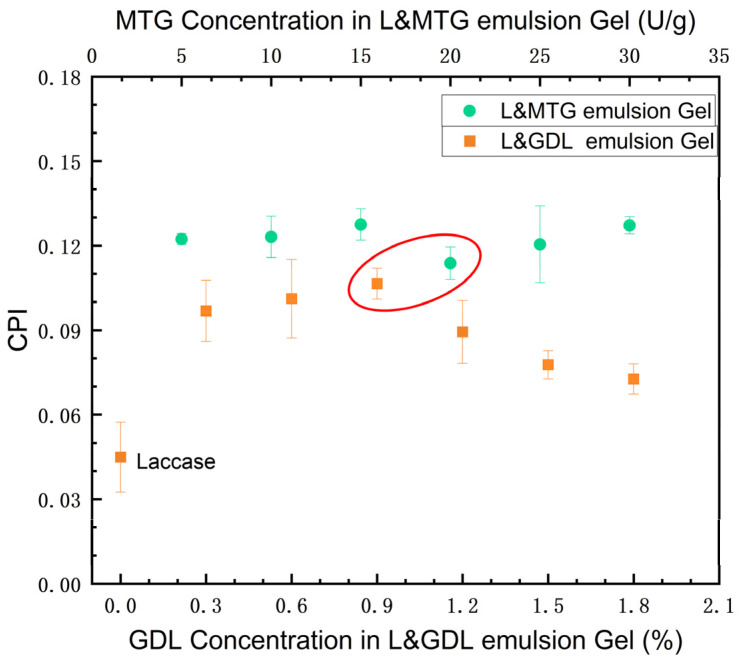
The CPI of SPI-SBP emulsion gels induced by laccase, L&GDL, and L&MTG. The plots of gels induced by laccase (0% GDL) and L&GDL were marked orange square (■) versus bottom X-axis, and the plots of gels induced by L&MTG were marked green circle (●) versus top X-axis.

Based on the Minkowski distance, the three-dimensional data of the gel textural properties were normalized into one-dimensional data, which was defined as CPI of the SPI-SBP emulsion gel (Figure 2). According to the Pearson correlation analysis, this CPI was positively correlated to hardness (r = 0.916), springiness (r = 0.9322), and cohesiveness (r = 0.9136). The positive correlation of gel hardness, springiness, and cohesiveness with CPI indicated the reliability of the regression model. Based on the normalized data, a regression analysis of hardness, springiness, and cohesiveness was performed, and the equation (Equation (5)) obtained was

(5) CPI=0.0926×H+0.0939×S+0.3199×C−0.5387

where H was the hardness of the gel; S was the springiness of the gel; C was the cohesiveness of the gel. R^2^ of the equation was 0.9785, which suggested that the linear regression model fitted the textural property data and could reflect the texture properties of the SPI-SBP emulsion gel.

Figure 2 shows CPI of emulsion gels under different induction methods, and the CPI value of the emulsion gel induced by L&MTG was generally higher than that of the gel induced by L&GDL. To analyze the effect of different induction methods on the SPI-SBP emulsion gel, induction combinations that yielded similar CPI were selected and marked with a red circle, as shown in Figure 2, for the subsequent studies. In the proceeding studies, the GDL concentration of the L&GDL SPI-SBP emulsion gel was set at 0.9%, and the MTG addition of the L&MTG SPI-SBP emulsion gel was set at 20 U/g.

### 3.3. Microstructure of the SPI-SBP Emulsion Gel

**Figure 3:**
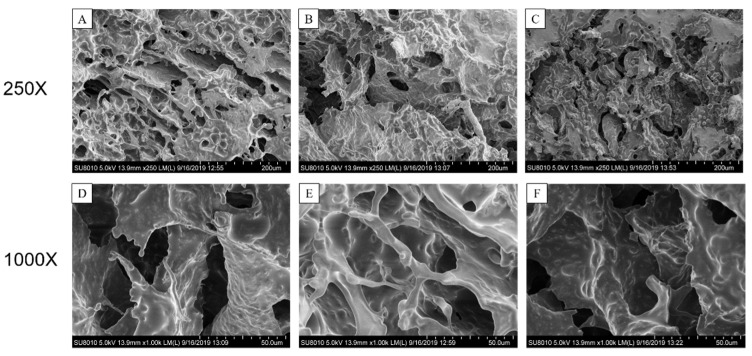
The SEM micrographs of SPI-SBP emulsion gels induced by laccase (**A**), L&GDL (**B**), and L&MTG (**C**) in 250×. (**D**–**F**) are a section of images (**A**–**C**) with higher magnification (1000×).

As shown in Figure 3, SEM was used to evaluate the effect of different induction methods on the microstructure of SPI-SBP emulsion gels. The SPI-SBP emulsion gel induced by laccase alone had a regular structure, consisting of uniform pores. The emulsion gels induced by L&GDL presented a porous structure, similar to the structure induced by laccase alone, yet the L&MTG emulsion gel yielded a denser structure with fewer pores, due to a higher degree of cross-linking. The SPI-SBP emulsion gel induced by laccase and thermal treatment has been reported to have a porous structure and the porosity value depended on SBP concentration [6]. Yan [13] also found that Corn fiber gum-SPI hydrogels induced by L&GDL exhibited a honeycomb network.

**Figure 4:**
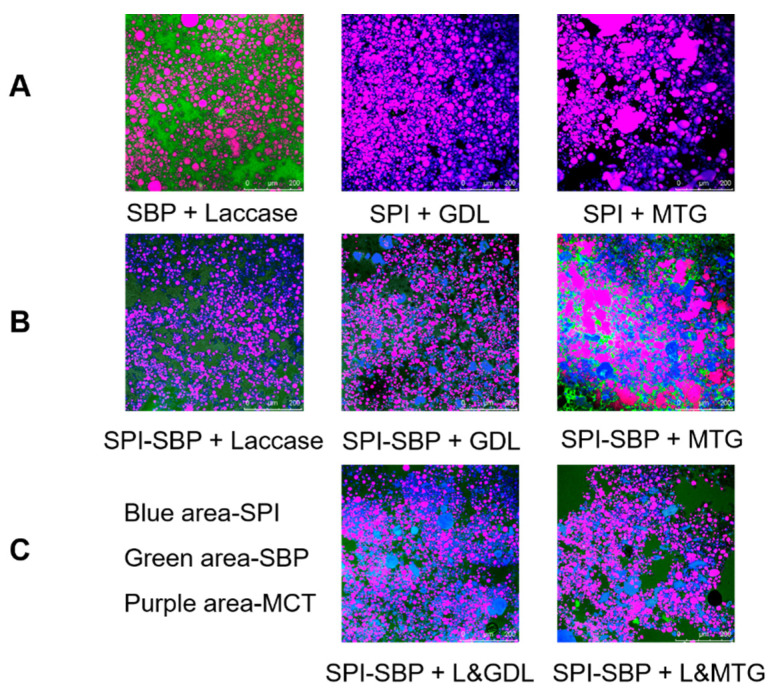
The CLSM micrographs of SPI or SBP emulsion gel induced by single method (**A**), SPI-SBP emulsion gel induced by single method (**B**), and SPI-SBP emulsion gel induced by double methods (**C**). The scale bar indicates 200 μM. SBP was labeled by FITC for green. SPI was stained by Rhodamine B for blue. MCT was stained by Nile Red for red.

In Figure 4, the green area of the CLSM result represents the FITC-labeled SBP, the blue area represents rhodamine-stained SPI, and the red area indicates Nile Red-stained MCT oil droplets (red oil droplets appear purple due to superposition with blue protein). As shown in Figure 4A, oil droplets were embedded in the networks of SPI or SBP. An aggregation of oil droplets occurred in the SPI emulsion gel induced by MTG alone, because MTG treatment increased the interfacial and surface tensions of proteins and the formation of a stronger interfacial gel network during gelation, which caused the rupture and re-aggregation of oil droplets [28]. In contrast, GDL can quickly disperse into the emulsion system and slowly release hydrogen ions to form a uniformed SPI network [29], so that the oil droplets can disperse uniformly in the network. Compared with the SPI and SPI-SBP emulsion gel induced by MTG alone in Figure 4A, B, the rupture and aggregation of oil droplets in the SPI-SBP emulsion gel induced by the duo-induction of L&MTG, presented in Figure 4C, was alleviated, indicating that the SBP network structure induced by laccase can support and protect the oil droplets to a certain extent, and reduce the damage to the oil droplets when MTG induces the SPI gel network. For L&GDL SPI-SBP emulsion gel, the internal matrix distribution of the emulsion gel induced by GDL was relatively uniform. SPI was in an aggregated state and dispersed within the network of SBP, and the oil droplets were embedded within the SPI and SBP network, which is in agreement with the previous study [6].

### 3.4. Swelling Properties of the SPI-SBP Emulsion Gel

**Figure 5:**
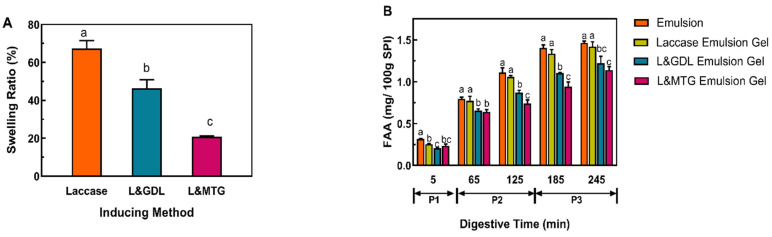

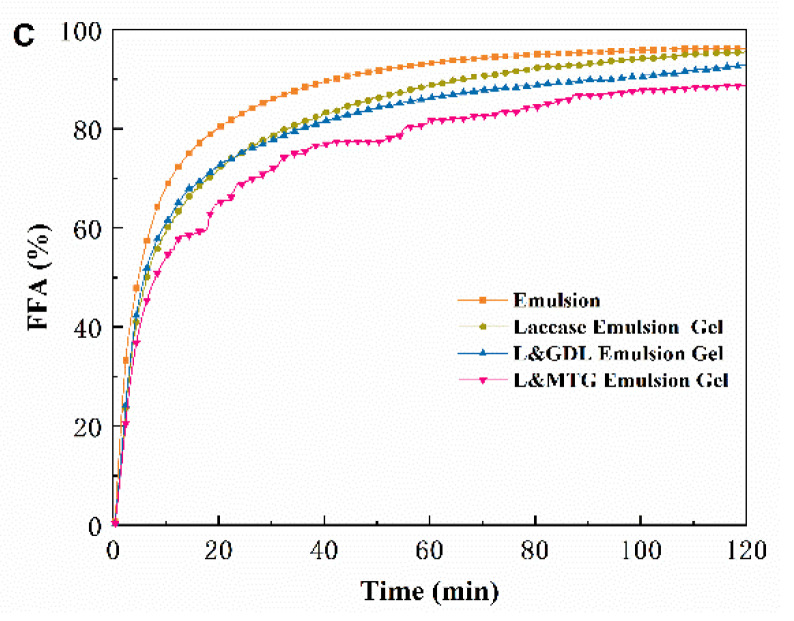
The swelling properties (**A**) and the digestive profiles of FAA (**B**) and FFA (**C**) for SPI-SBP emulsion gels induced by different methods. Different letters indicate significant differences in the same group at *p* < 0.05 level. P1: Oral Phase; P2: Gastric Phase; P3: Intestinal Phase.

A previous study indicated that the greater the swelling, the easier it is for water and digestive enzymes to diffuse into the gel network structure, which in turn promotes the release of functional factors [20]. As shown in Figure 5A, compared with the SPI-SBP emulsion gel induced by laccase alone, the additional inducing agent reduced the swelling of the emulsion gels. For the duo-inducing SPI-SBP emulsion gel, the swelling ratio of the L&MTG emulsion gel was lower than the L&GDL emulsion gel, which mainly depends on the microstructure. When pore size in the hydrogel network becomes smaller, the free movement of molecular chains is restricted. Then, the interaction between the hydrophobic groups inside the hydrogel increases, which makes it difficult for water molecules to enter the hydrogel network [30]. Based on the SEM results, the L&MTG emulsion gel had a denser structure, with a higher degree of cross-linking and fewer pores, so its swelling ratio was lower than others. On the other hand, gels that have a loose network structure display better swelling abilities, which was the case in L&GDL induced emulsion gels.

### 3.5. The Digestion Profiles of the SPI-SBP Emulsion Gel

As shown in Figure 5B, FAA released during digestion increased gradually in all samples. During the early stage of the gastric digestion phase (P2, 5–65min), the L&MTG emulsion gel showed that a larger amount of FAA released. Because of the dense structure, the L&MTG emulsion gel should have a lower degree of hydrolysis and FAA release rate than L&GDL emulsion gel during digestion; however, the results showed that was released at a greater rate for L&MTG in the oral digestion phase (P1) until the middle of P2. This is because compared with GDL induction, gelation by MTG resulted in more uncross-linked proteins [22], which were digested more easily than the proteins cross-linked in the gel network. At the end of P2 (125 min), the L&MTG emulsion gel began to present its advantage of having a denser structure, which resulted in releasing a lower amount of FAA. In this phase, the protein that was most hydrolyzed was the cross-linked one in the gel network, and the denser structure of the gel network led to the lower rate of hydrolyzation for cross-linked proteins [31]. At the end of the intestinal phase (P3), the order of the FAA release amount from highest to lowest was as follows: the emulsion, the emulsion gel induced by laccase alone, L&GDL emulsion gel, and L&MTG emulsion gel. Out of the three induction methods, the L&MTG emulsion gel had the lowest FAA release, due to the lowest swelling ratio with small pore size, preventing the digestive enzymes from entering into the gel network and imparting the effect of enzymolysis [32].

After experiencing shear effects in the mouth and stomach, the oil in the emulsion is mixed with pancreatic lipase, trypsin, bile salts, and phospholipids in the small intestine. Oil digestion under the action of lipase was mainly carried out in the small intestine [33], so the release rate of FFA from SPI-SBP emulsion gels was analyzed in SIF. As can be observed in Figure 5C, the release rate of FFA from emulsion gels presented a rapid initial release from 0 min to 20 min, before plateauing at 60 min. At the end of the intestinal digestion process (120 min), the FFA release rate of each sample from high to low was as follows: SPI-SBP emulsion, the emulsion gel induced by laccase alone, L&GDL emulsion gel, L&MTG emulsion gel. In the SPI-SBP emulsion gel system, the rate and extent of oil digestion usually depend on the integrity of network structure [34]. In the duo-inducing SPI-SBP emulsion gels, the SPI network was digested during the digestive process, and the digestive rate of the SPI network in the L&GDL emulsion gel was higher than that in the L&MTG emulsion gel, based on the results of the amount of FAA released. On the other hand, the SBP network in L&GDL emulsion gels remained and protected the oil droplets from being digested. SPI-SBP emulsion did not form any network, thus, the amount of FFA released from L&GDL emulsion gel was higher than that from the L&MTG emulsion gel but still lower than the other two at the end of digestion.

### 3.6. EE of the SPI-SBP Emulsion Gel as the Multi-Phased Nutrients Carrier

**Figure 6:**
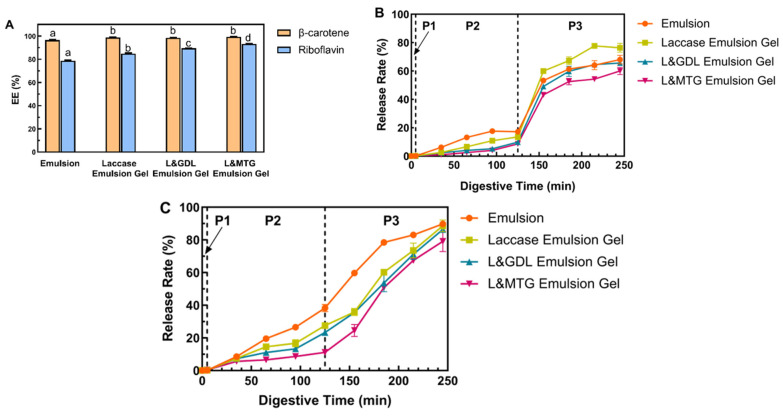
The embedding (**A**) and the release profiles of β-carotene (**B**) and riboflavin (**C**) from co-loading SPI-SBP emulsion gel induced by different methods. Different letters indicated significant differences among different inducing methods at *p* < 0.05 level. P1: Oral Phase; P2: Gastric Phase; P3: Intestinal Phase.

EE is essential to the performance of the nutrient delivery system, and affects the storage and bioavailability of functional factors that are embedded in the delivery system [35]. In this study, the hydrophilic phase (SPI-SBP network) was loaded with the riboflavin, and the lipophilic phase (MCT) was loaded with β-carotene. As indicated in Figure 6A, there was no significant difference in the EE of β-carotene between the laccase emulsion gel and duo-induced emulsion gels. The oil droplets containing β-carotene were wrapped in the gel network, which reduced direct contact of the oil droplets with the outside environment [36]. For the riboflavin embedded in the water phase, compared with the SPI-SBP emulsion, the emulsion gel had higher EE of riboflavin. The order of EE for riboflavin from low to high was as follows: the emulsion, the emulsion gel induced by laccase alone, L&GDL emulsion gel, and L&MTG emulsion gel. The EE of hydrophilic functional factors is related to the porosity of the gel matrix [37]. Combined with the results from previously stated microstructure and swelling ratio measurement, the SPI-SBP emulsion gel that had a looser and more porous structure tended to have lower EE of riboflavin.

### 3.7. The Release Profiles of Co-Encapsulated Functional Factors

The release of β-carotene in the SPI-SBP emulsion gel during the digestion process is mainly related to the digestion process of oil embedded in the gel network [38]. After simulated gastric digestion, these oil droplets were released partly into the SGF from the gel network. The part of β-carotene which was measured was in the free oil droplets, but those oil droplets did not undergo continued digestion in SSF and SGF [39]. As shown in Figure 6B, during P1, there was no significant release of β-carotene, as observed in all samples. As the digestion proceeded to P2, the release rate of β-carotene from the emulsion began to increase significantly compared with the emulsion gels, which could indicate that the gel carrier presented in this study was capable of delivering and control-releasing β-carotene in vitro simulated digestion. After entering P3, the release rate of β-carotene in each sample greatly increased, mainly due to the action of the pancreatic lipase in the intestinal phase. Lipase can hydrolyze the oil droplets in the emulsion gel, leading to the release of β-carotene from the oil phase [20]. At the end of P3 (245 min), the β-carotene release rate from both L&MTG (60.1%) and the L&GDL (65.7%) emulsion gel was lower than that of the other systems (68.0% and 76.4%). In the SPI-SBP emulsion gels induced by duo-induction methods, the SPI network was digested by pepsin and trypsin during the digestive process, while due to lack of digestive enzymes, the SBP network continued to reduce the contact between oil droplets and lipase, protecting β-carotene from being released [19]. Finally, the β-carotene in all three emulsion gels (laccase, L&GDL, and L&MTG) was still partially wrapped in the gel network after 245 min (P3), indicating that the gel carrier required more time to release the residual β-carotene [40]. Thus, the releasing process of β-carotene was more closely related to the polysaccharide network structure in the system.

As indicated in Figure 6C, during P1, no significant release of hydrophilic riboflavin was observed in all samples. After 65 min, the SPI-SBP emulsion and the emulsion gel induced by laccase alone had released more riboflavin. When samples entered P3, all the release rates of riboflavin increased as the gel network was further hydrolyzed. At the end of P3 (245 min), the riboflavin release rate of each SPI-SBP sample from high to low was as follows: SPI-SBP emulsion (89.8%), the emulsion gel induced by laccase alone (88.6%), L&GDL emulsion gel (86.4%), L&MTG emulsion gel (79.1%). The L&MTG emulsion gel had the lowest riboflavin release rate at each digestion phase, because the L&MTG emulsion gel had a dense structure with the lowest swelling ratio, which controlled the riboflavin release [41]. Therefore, the release profiles of riboflavin had a higher correlation with the swelling performance of the gel and the density of the gel network structure. The looser the network structure, the easier it was for the embedded functional factors to be released through water diffusion.

## 4. Conclusions

In this study, a novel SPI-SBP emulsion gel was prepared as a multi-phased carrier to co-encapsulate both hydrophilic and lipophilic nutrients. Compared with the L&GDL emulsion gel, the structure of the L&MTG emulsion gel was denser and had a lower swelling ratio; therefore, the digestion rate was lower. The EE of β-carotene in the SPI-SBP emulsion gel was not affected by the induction method, while the EE of riboflavin was mainly correlated to the pore size of the SPI-SBP network synthesized under different induction methods. During simulated digestion studies, the release of both β-carotene and riboflavin in the SPI-SBP emulsion gels was regulated by the gel network induced by different induction methods. The swelling ratio accompanied by these structures was responsible for the controlled-release profiles of functional factors co-loaded within the system. Therefore, the capability of SPI-SBP emulsion gels of encapsulating multiple functional factors with different physicochemical properties will further expand their applications in the food industry.

## Data Availability

Not applicable.

## Supplementary Materials

The following supporting information can be downloaded at: https://www.mdpi.com/article/10.3390/foods11030469/s1. Figure S1: Effects of GDL concentration on pH changes of SPI-SBP emulsion.
