# Supplementary material for: Preparation and Characterization of a Novel Soy Protein Isolate-Sugar Beet Pectin Emulsion Gel and Its Application as a Multi-Phased Nutrient Carrier
Source: Foods. 2022 Feb 5;11(3):469. doi: 10.3390/foods11030469 (PMC8833956; doi:10.3390/foods11030469)
Supplement: Supplementary file 1 [file foods-11-00469-s001.zip › foods-1569528-supplementary.pdf]

foods MDPI

*Supplementary materials*

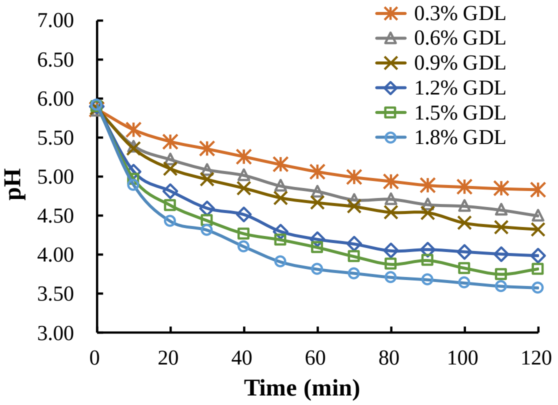


**Figure S1.** Effects of GDL concentration on pH changes of SPI-SBP emulsion.
